# Supplementary material for: Causal Relationship between Aging and Anorexia Nervosa: A White-Matter-Microstructure-Mediated Mendelian Randomization Analysis
Source: Biomedicines. 2024 Aug 16;12(8):1874. doi: 10.3390/biomedicines12081874 (PMC11351342; doi:10.3390/biomedicines12081874)
Supplement: Supplementary file 1 [file biomedicines-12-01874-s001.zip › Supplementary Figure/Figure S4/Singe SNP plot of MR analysis for TL to ubm-b-2033.pdf]

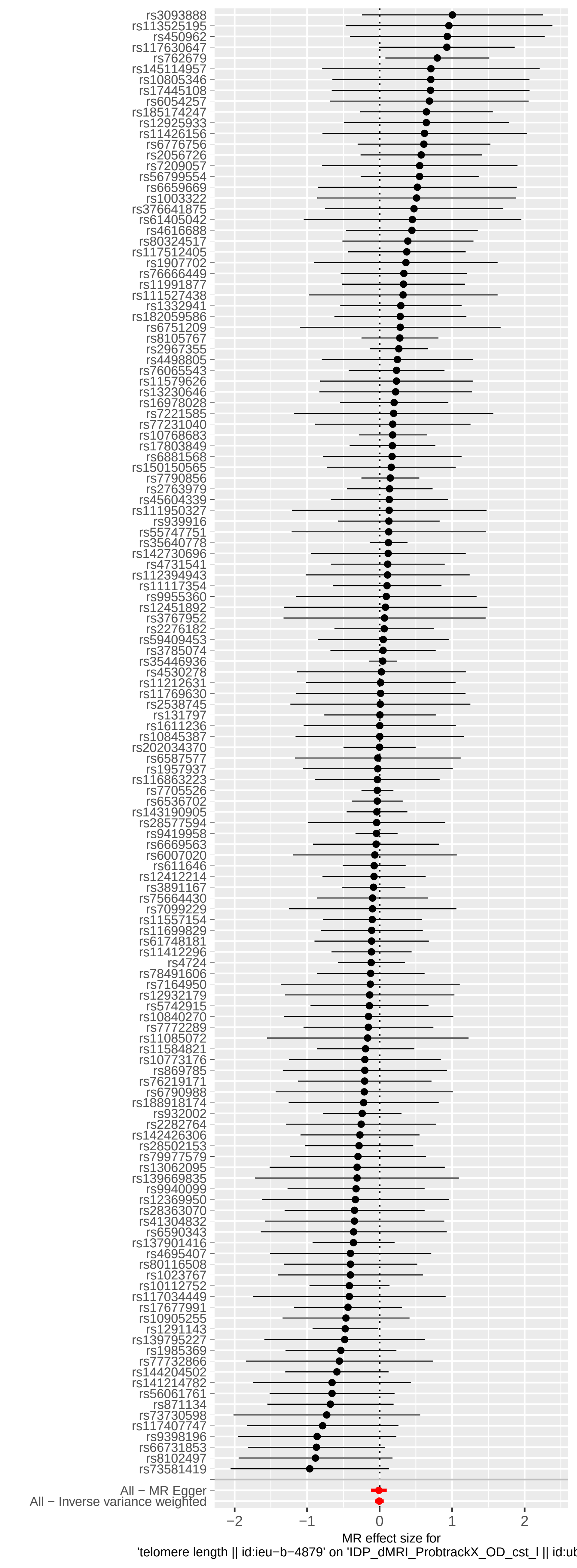
rs3093888
rs113525195
rs450962
rs117630647
rs762679
rs145114957
rs10805346
rs17445108
rs6054257
rs185174247
rs12925933
rs11426156
rs6776756
rs2056726
rs7209057
rs56799554
rs6659669
rs1003322
rs376641875
rs61405042
rs4616688
rs80324517
rs117512405
rs1907702
rs76666449
rs11991877
rs111527438
rs1332941
rs182059586
rs6751209
rs8105767
rs2967355
rs4498805
rs76065543
rs11579626
rs13230646
rs16978028
rs7221585
rs77231040
rs10768683
rs17803849
rs6881568
rs150150565
rs7790856
rs2763979
rs45604339
rs111950327
rs939916
rs55747751
rs35640778
rs142730696
rs4731541
rs112394943
rs11117354
rs9955360
rs12451892
rs3767952
rs2276182
rs59409453
rs3785074
rs35446936
rs4530278
rs11212631
rs11769630
rs2538745
rs131797
rs1611236
rs10845387
rs202034370
rs6587577
rs1957937
rs116863223
rs7705526
rs6536702
rs143190905
rs28577594
rs9419958
rs6669563
rs6007020
rs611646
rs12412214
rs3891167
rs75664430
rs7099229
rs11557154
rs11699829
rs61748181
rs11412296
rs4724
rs78491606
rs7164950
rs12932179
rs5742915
rs10840270
rs7772289
rs11085072
rs11584821
rs10773176
rs869785
rs76219171
rs6790988
rs188918174
rs932002
rs2282764
rs142426306
rs28502153
rs79977579
rs13062095
rs139669835
rs9940099
rs12369950
rs28363070
rs41304832
rs6590343
rs137901416
rs4695407
rs80116508
rs1023767
rs10112752
rs117034449
rs17677991
rs10905255
rs1291143
rs139795227
rs1985369
rs77732866
rs144204502
rs141214782
rs56061761
rs871134
rs73730598
rs117407747
rs9398196
rs66731853
rs8102497
rs73581419
All − MR Egger
All − Inverse variance weighted
−2
−1
0
1
2
MR effect size for
'telomere length || id:ieu−b−4879' on 'IDP_dMRI_ProbtrackX_OD_cst_l || id:ub
